# Noise decouples covariances from interaction strength

**DOI:** 10.1186/1471-2202-14-S1-P164

**Published:** 2013-07-08

**Authors:** D Grytskyy, T Tetzlaff, M Diesmann, M Helias

**Affiliations:** 1 Inst. of Neuroscience and Medicine (INM-6) and Inst. for Advanced Simulation (IAS-6), Jülich Research Centre and JARA, Jülich, Germany; 2 Medical Faculty, RWTH Aachen, Aachen, Germany

## 

**Figure 1:**
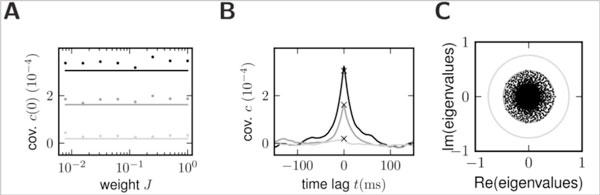
**A Zero time-lag cross covariance averaged over pairs of excitatory (black) and inhibitory (light gray) cells and of one excitatory and one inhibitory neuron (gray) in simulation (dots) and theory (lines)**. **B **Cross covariance functions averaged as in A (same gray code) obtained from simulations at one coupling strength. Crosses show the analytical prediction. **C **Set of eigenvalues of a random connectivity matrix after linearization (black dots) with the corresponding spectral radius (gray circle) and the maximum radius for any synaptic strength (light gray circle).

Correlated neural activity is a known feature of the brain [2] and evidence increases that it is closely linked to information processing [1]. In our recent work we have shown how to map different network models, including binary networks, onto linear dynamics [4]. For binary neurons the mean-field approach takes random fluctuations into account to accurately predict the average activity in such networks [5]. Expressions for covariances follow from a master equation [3]. Binary neurons with a Heaviside gain function are inaccessible to the classical treatment [3]. Based on our earlier preliminary results [6] here we show how random fluctuations generated by the network effectively linearize the system of binary neurons, including the case of the Heaviside gain function, and how they implement a self-regulating mechanism which renders population-averaged covariances independent of the synaptic coupling strength. Figure 1A, B illustrate this invariance.

The mechanism is based on the increase of fluctuations in the input signal in proportion to the synaptic weight. The fluctuations cause portions of the gain function with smaller slope to be visited more frequently, effectively reducing the transmission gain. This keeps the linearized system away from instability, with the eigenvalues of its effective connectivity matrix bounded by a constant less than unity (see Figure 1C). Although of local origin the mechanism controls global features of the network dynamics.
